# Endoscopic stapedotomy: classic versus reversal technique

**DOI:** 10.1007/s00405-023-07880-7

**Published:** 2023-02-17

**Authors:** Waleed Moneir, Yasser Wafeek Khafagy, Nada Nagah Salem, Ahmed Hemdan

**Affiliations:** Department of Otolaryngology, Faculty of Medicine, Mansoura University, El-Gomhoria Street, Mansoura, Egypt

**Keywords:** Stapedotomy, Endoscopic, Classic, Reversal

## Abstract

**Objectives:**

To compare hearing outcome and surgical complications between endoscopic classic and reversal stapedotomies.

**Patients and methods:**

A prospective single blinded randomized clinical study carried out on 60 patients with otosclerosis who were randomized into two groups; each containing 30 patients. Patients in group 1 underwent endoscopic classic stapedotomy. Patients in group 2 underwent endoscopic reversal stapedotomy. Both groups were compared as regards hearing outcome and surgical complications.

**Results:**

The difference in the hearing outcome between the two groups was statistically non-significant. Post-operative closure of the air bone gap (ABG) within 10 dB was attained in 76.67% and 80% of patients in groups 1 and 2, respectively. The differences in the surgical complications between the two studied groups were statistically non-significant.

**Conclusion:**

Endoscopic classic and reversal stapedotomies are comparable to each other as regards hearing outcome and surgical complications. The authors recommend further studies with relatively larger sample size.

## Introduction

Otosclerosis is a remodeling disorder of the otic capsule where foci of resorbed bone were present, associated with newly formed bone, together with vascular proliferation [1]. Progressive hearing loss is the main presentation and stapes surgery; either stapedectomy or stapedotomy; is the primary treatment [2, 3]. Although some authors observed similar results with both methods (stapedectomy and stapedotomy), the current tendency is to replace stapedectomy with stapedotomy due to greater air bone gap (ABG) closure in high frequencies together with higher speech discrimination scores after surgery [4–6]. The order of the surgical steps during stapes surgery is another crucial factor. Accordingly, two techniques can be performed: classic and reversal techniques. The classic technique starts with removal of the stapes superstructure, followed by perforation of the stapes footplate, and ends with insertion of the Teflon piston. The reversal technique; on the other hand; begins with footplate perforation, then insertion of the Teflon piston, and ends with removal of the superstructure [7]. Throughout the literatures, many studies were conducted to compare classic and reversal stapedotomies. However, all these studies were carried out using the microscope. As far as we know, our current study is the first in the literatures that compared such two techniques endoscopically.

## Patients and methods

This study was a prospective single blinded randomized clinical study carried out between September 2021 and September 2022. Approval from Institutional ethics committee was obtained (code: MS.21.11.1758). The study was conducted among 60 patients with clinical evidence of otosclerosis who were randomly divided into two equal groups (each containing 30 patients) using computer generated block randomization. Patients in group 1 underwent endoscopic classic stapedotomy while patients in group 2; on the other hand; underwent endoscopic reversal stapedotomy.

All involved patients had clinically evidenced otosclerosis including gradual progressive diminution of hearing with intact tympanic membrane, an audiogram showing ABG > 20 dB at the frequencies of 0.5, 1, 2 and 3 kHz together with absent stapedial reflex. Revision cases, cases with obliterative type of otosclerosis, and cases with facial nerve dehiscence were all excluded. Since narrow footplate is a contraindication for reversal stapedotomy, we also excluded all cases with narrow footplate to make the whole patients amenable for both classic and reversal stapedotomies. We defined narrow footplate as a footplate that could not accommodate a 0.7 mm manual perforator.

A 0° endoscope, 17 cm length and 4 mm diameter (Karl Storz, Germany) which was coupled to a high-definition (HD) camera head connected with a monitor (Karl Storz, Germany) were used. All surgeries were performed under local anesthesia (2% lidocaine with 1:50,000 epinephrine). The surgery began with 4 quadrant injection of the local anesthetic solution. Incision of the skin of the external ear canal was performed followed by tympano-meatal flap elevation towards the tympanic annulus. Entry to the middle ear was then carried out by raising of the tympanic annulus out of its sulcus. Curettage of the posterosuperior canal wall was carried out till the following structures were fully exposed: the stapes footplate, the facial nerve, the stapedial tendon and the pyramid. The ossicular chain mobility was then assessed to confirm the true diagnosis of otosclerosis. Measurement of the size of footplate was then done by using a 0.7 mm manual perforator. Cases where the footplate could not accommodate the 0.7 mm perforator were excluded because this would be a narrow footplate which is a contraindication for reversal stapedotomy.

### Group 1

**Fig. 1:**
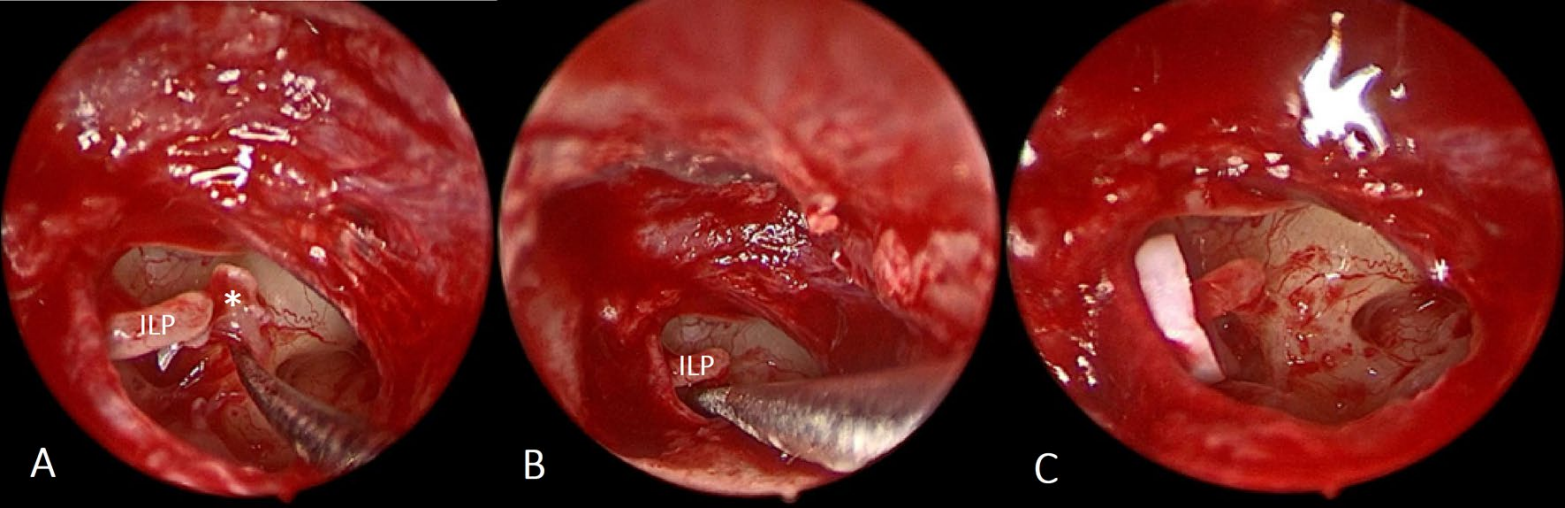
Steps of endoscopic classic stapedotomy: **A** Removal of stapes superstructure. **B** Perforation of the stapes footplate. **C** Insertion of the Teflon piston. (*Stapes superstructure, ILP incus long process)

Classic stapedotomy was done starting by removal of the stapes superstructure (Fig. 1A). Using 0.7 mm manual perforator, perforation of the stapes footplate was then performed (Fig. 1B). The perforation was done under vision in alternating clockwise and counterclockwise rotatory movement and without pushing. A Teflon piston (0.6 mm in diameter) was inserted and fixed to the incus long process (Fig. 1C). The tympano-meatal flap was then repositioned and supported with small pieces of Gelfoam^®^.

### Group 2

**Fig. 2:**
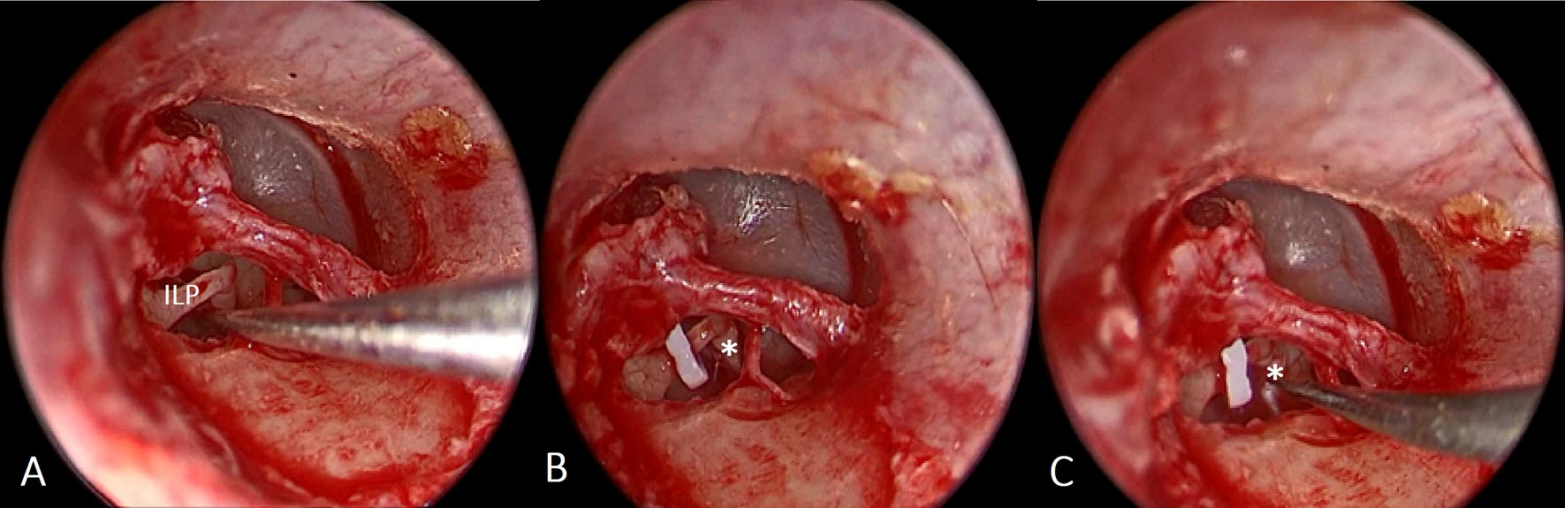
Steps of endoscopic reversal stapedotomy: **A** Perforation of the stapes footplate. **B** Insertion of the Teflon piston. **C** Removal of stapes superstructure. (*Stapes superstructure, ILP incus long process)

Reversal stapedotomy was performed which began by perforation of the footplate (Fig. 2A) using a 0.7 mm manual perforator under vision in alternating clockwise and counterclockwise rotatory movement and without pushing. This was followed by insertion of 0.6 mm in diameter Teflon piston (Fig. 2B). Finally, removal of the stapes superstructure was done (Fig. 2C). The tympano-meatal flap was then repositioned and supported with small pieces of Gelfoam®.

### Follow-up

The initial scheduled visit for follow-up was carried out 1 week after surgery where the covering gauze was removed, and antibiotic ear drops were prescribed for 1 week. Audiological assessment was done 3 months after surgery in line with the guidelines of the American Academy of Otolaryngology- Head and Neck Surgery Committee on Hearing and Equilibrium. Pre- and post-operative air conduction (AC) thresholds and bone conduction (BC) thresholds were estimated at the frequencies 0.5, 1, 2, and 3 kHz. To calculate the 3 kHz results, the results for 2 and 4 kHz had to be averaged. The air bone gap (ABG) was estimated by calculating the difference between AC and BC thresholds [8].

### Data collection and statistical analysis

Pre- and post-operative data were collected, tabulated, and analyzed. The data were presented in the form of mean ± standard deviation (SD). Analysis was done using SPSS for Windows version 28. statistical software program (Statistical Package for Social Sciences = SPSS Inc., Chicago, IL, USA). The paired *t* test was used for comparison of the pre-operative with the post-operative results. The chi-square test was utilized to compare the results of the two groups. A statistically significant result was considered when *P* value was < 0.05.

## Results

Sixty patients were involved in this study. Group 1 comprised 30 patients: 19 females (63.33%) and 11 males (36.67%). Their mean age ± SD was 41.43 ± 10.76 years. Group 2 consisted of 30 patients: 21 females (70%) and 9 males (30%). Their mean age ± SD was 44.33 ± 8.14 years. Statistically non-significant differences in both sex and age between the two groups were found (*P* values for differences in sex and age between the two groups were 0.49 and 0.16 respectively).

Table 1 shows the pre- and post-operative hearing results in groups 1 and 2. The mean pre-operative AC thresholds in groups 1 and 2 were 56.21 ± 11.51 and 52.00 ± 10.47, respectively. Such difference was statistically non-significant (*P* value 0.14). The mean post-operative AC thresholds in groups 1 and 2 were 30.35 ± 14.22 and 27.82 ± 12.25 respectively, with statistically non-significant difference (*P* value 0.47). In both groups, statistically significant reductions in post-operative AC thresholds in comparison to the pre-operative values were reported (*P* value < 0.001 in both groups). The mean pre-operative BC thresholds in groups 1 and 2 were 23.54 ± 7.34 and 22.32 ± 4.73, respectively. Such difference was statistically non-significant (*P* value 0.43). The mean post-operative BC thresholds in groups 1 and 2 were 21.50 ± 12.27 and 20.17 ± 9.87 respectively, with statistically non-significant difference (*P* value 0.66). In both groups, no statistically significant differences between pre-operative and post-operative BC thresholds were reported (*P* values for groups 1 and 2 were 0.36 and 0.16, respectively). The mean pre-operative ABG thresholds in groups 1 and 2 were 32.67 ± 7.33 and 29.68 ± 8.40, respectively. Such difference was statistically non-significant (*P* value 0.15). The mean post-operative ABG thresholds in groups 1 and 2 were 8.92 ± 5.28 and 7.61 ± 5.52 respectively, with statistically non-significant difference (*P* value 0.29). In both groups, statistically significant reductions in post-operative ABG thresholds in comparison to the pre-operative values were reported (*P* value < 0.001 in both groups). The mean pre-operative speech reception thresholds (SRTs) in groups 1 and 2 were 55.67 ± 9.80 and 51.83 ± 6.88, respectively. Such difference was statistically non-significant (*P* value 0.08). The mean post-operative SRTs in groups 1 and 2 were 29.50 ± 12.82 and 24.83 ± 11.56 respectively, with statistically non-significant difference (*P* value 0.14). In both groups, statistically significant reductions in post-operative SRTs in comparison to the pre-operative values were reported (*P* value < 0.001 in both groups). The mean pre-operative speech discrimination scores (SDSs) in groups 1 and 2 were 95.73 ± 7.12 and 97.47 ± 3.28, respectively. Such difference was statistically non-significant (*P* value 0.26). The mean post-operative SDSs in groups 1 and 2 were 96.13 ± 7.08 and 97.67 ± 3.16 respectively, with statistically non-significant difference (*P* value 0.31). In both groups, no statistically significant differences between pre-operative and post-operative SDSs were reported (*P* values in groups 1 and 2 were 0.08 and 0.18, respectively).

**Table 1:** Comparison between groups 1 and 2 as regard mean AC, BC, ABG, SRT and SDS

	Group 1	Group 2	*P* value
AC threshold (mean ± SD)			
Preoperative	56.21 ± 11.51	52.00 ± 10.47	0.14
Postoperative	30.35 ± 14.22	27.82 ± 12.25	0.47
* P* value	< 0.001	< 0.001	
BC threshold (mean ± SD)			
Preoperative	23.54 ± 7.34	22.32 ± 4.73	0.43
Postoperative	21.50 ± 12.27	20.17 ± 9.87	0.66
* P* value	0.36	0.16	
ABG (mean ± SD)			
Preoperative	32.67 ± 7.33	29.68 ± 8.40	0.15
Postoperative	8.92 ± 5.28	7.61 ± 5.52	0.29
* P* value	< 0.001	< 0.001	
SRT (mean ± SD)			
Preoperative	55.67 ± 9.80	51.83 ± 6.88	0.08
Postoperative	29.50 ± 12.82	24.83 ± 11.56	0.14
* P* value	< 0.001	< 0.001	
SDS (mean ± SD)			
Preoperative	95.73 ± 7.12	97.47 ± 3.28	0.26
Postoperative	96.13 ± 7.08	97.67 ± 3.16	0.31
* P* value	0.08	0.18	

Post-operative closure of the ABG within 10 dB was attained in 23 cases in group 1 (76.67%) and in 24 cases in group 2 (80%). Such difference was statistically non-significant (*P* value 0.77).

Table 2 shows the incidence of complications among both groups. Neither sensory neural hearing loss nor floating footplate were reported in the two groups. A tear in the tympanic membrane occurred in only 1 case in group 1 while in group 2, no tears were encountered. Such difference in the incidence of the tears in the tympanic membrane was statistically non-significant (*P* value 0.33). Incus subluxation occurred in 2 cases in group 1 (6.66%) and 1 case in group 2 (3.33%) with statistically non-significant difference (*P* value 0.33). Vertigo was encountered in 6 cases (20.00%) in group 1 and in 4 cases (13.33%) in group 2 with statistically non-significant difference (*P* value 0.16). Taste disturbance reported in 6 cases (20.00%) in group 1 and in 3 cases (10.00%) in group 2 with statistically non-significant difference (*P* value 0.08).

**Table 2:** Difference in complications between the 2 groups:

	Group 1	Group 2	*P* value
Sensory neural hearing loss	0 (0%)	0 (0%)	–
Floating footplate	0 (0%)	0 (0%)	–
Tympanic membrane tear	1 (3.33%)	0 (0%)	0.33
Incus subluxation	2 (6.66%)	1 (3.33%)	0.33
Vertigo	6 (20.00%)	4 (13.33%)	0.16
Taste disturbance	6 (20.00%)	3 (10.00%)	0.08

## Discussion

Surgical techniques for treatment of otosclerosis have been improved, updated, or adjusted over time to lower intraoperative and post-operative problems and boost overall effectiveness [9]. The order of surgical steps had been a matter of debate. Early cases were treated with classic stapedotomy, where the stapes superstructure was first removed, the footplate was then perforated, and lastly the prosthesis was fixed to the incus. Fisch provided a different sequence of the surgical steps where he first performed perforation of the stapes footplate, then removed the stapes superstructure and finally inserted the prosthesis. Then he shifted towards a whole reversal of the classic stapedotomy steps where he started by perforation of the stapes footplate, then inserted the prosthesis and in the end removed the stapes superstructure [7].

Not only the order of surgical steps was debatable but also the surgical tool, whether to use the microscope or the endoscope. Different studies compared microscopic with endoscopic stapedotomy. Although hearing outcome and post-operative complications were comparable, the endoscope gives a very important advantage which is the excellent visualization [10–14].

The utilization of endoscope in stapes surgery has many advantages. The wide angle of view offered by endoscope allows better exposure of both stapes and its footplate. In addition, it facilitates detection of any anatomical variations or pathological changes. Moreover, it allows confirmation of the proper insertion of the prosthesis [15, 16]. Despite these advantages, endoscope also has many disadvantages. One of these disadvantages is the single-handed surgery as the other hand is occupied the endoscope. In addition, endoscope appeared to enhance exposure not necessarily visualization. Moreover, the incidence of perforation of the tympanic membrane during flap elevation; using the endoscope; was high. Furthermore, the incidence of chorda tympani injury with subsequent dysgeusia was high when the endoscope was used in stapes surgery [17]. As in every modern surgical technique, endoscopic stapes surgery has a learning curve that should be overcome. Such learning curve is longer for endoscopic than microscopic ear surgery [18–20] making it the main cause that dampen most otologists from starting endoscopic stapedotomy [20]. In addition, the depth of perception is reduced when the endoscope is used. Such problem can be overcome by changing the viewpoint while moving the endoscope and the surgical instruments in the field. In addition, the progress in the learning curve converts this problem into a minor one [18, 21].

Throughout the literatures, many studies were conducted to compare classic and reversal stapedotomies. All these studies were performed using the microscope. To our knowledge, our current study is the first in the literatures that compared classic and reversal stapedotomies using the endoscope.

In our study, statistically significant reductions in post-operative AC thresholds, ABGs and SRTs were obtained in both groups indicating that both techniques were effective in management of associated hearing loss. When both groups were compared, no statistically significant differences in post-operative AC thresholds, ABGs or SRTs were found. Moreover, closure of the ABG within 10 dB was attained in 76.67% and 80% of cases in groups 1 and 2 respectively, with statistically non- significant difference. All these findings suggested that endoscopic classic and reversal techniques were comparable as regards hearing outcome. Such finding was in agreement with the previously published studies [22–27].

Sensorineural hearing loss is relatively uncommon complication [28]. It is believed to occur due injury of the inner ear either during perforation or during insertion of the prosthesis. When the technical fault is ruled out, other causes are conceivable including reparative granuloma, labyrinthitis, and perilymph fistula [29].

During stapes surgery, small perforations of the tympanic membrane are usually closed with fascial graft at the end of the surgery with excellent result. Injury of the chorda tympani typically occurs during curettage or during its mobilization to expose the oval window. Such complication was estimated to occur in about 3% of cases in the literatures [30, 31]. House proposed that division of the chorda tympani; in comparison to stretching and manipulation; gives milder symptoms [32]. Mahendran et al., on the contrary, did not agree with these results. They evaluated the impacts of cutting and manipulation of the chorda tympani and presumed that whenever possible, the chorda tympani would be preserved since its cutting ends in significantly worse symptoms than its manipulation [31].

In our study, no sensory neural hearing loss was encountered in any of the cases of both groups. In addition, no statistically significant differences were reported between the two groups as regards post-operative vertigo and taste disturbance. All these findings were comparable to the previously published studies [22–27].

Floating footplate is one of the major complications that may be encountered during stapes surgery. In this condition, the footplate is accidently mobilized and may be pushed into the vestibule. Such complication may occur when the footplate is pushed too strong with manual perforator or even with microdrill. To avoid this complication, perforation should be done before removal of the stapes superstructure (as in reversal stapedotomy). Such support provided by the stapes superstructure will prevent footplate mobilization during perforation. Another method of prevention of such complication was the utilization of CO_2_ laser in the perforation, which enables the surgeon to create a perforation in the footplate without pushing on the footplate [33]. Accordingly, it is unsurprising to find in the literatures that reversal stapedotomy or the utilization of CO_2_ laser are the best ways to prevent such complication [22–27]. In our study, on the other hand, we did not encounter floating footplate in any of the cases of both groups. This was because alternating clockwise and counterclockwise rotatory movement without pushing will perforate rather than push the footplate. In addition, the excellent visualization provided by the endoscope allowed us to see and feel the perforation making it controlled perforation. On the hand, during microscopic stapedotomy we usually feel the perforation more than we see it except after its creation, so the perforation is usually uncontrolled with liability for pushing that may result in floating footplate. Accordingly, we can say that endoscopic stapedotomy together with alternating clockwise and counterclockwise rotatory perforation without pushing can prevent floating footplate.

Incus subluxation was another complication that may be encountered during stapes surgery. It was reported to occur if removal of stapes superstructure was done before insertion of the prosthesis. Thus, it is also unsurprising to encounter such complication in classic stapedotomy than in reversal type [15, 16, 18, 19, 21, 22]. In our study, on the other hand, no significant difference in the incidence of incus subluxation was reported between the two groups. This may indicate that the utilization of the endoscope may reduce the incidence of incus subluxation during classic stapedotomy. This may be explained by the fact that better visualization (offered by the endoscope) allows smooth insertion of the prosthesis without excessive manipulation that may cause incus subluxation. Such finding gives an advantage for the endoscope over the microscope during stapes surgery.

Since this study; to our knowledge; is the first randomized controlled trial that compared classic and reversal stapedotomy techniques endoscopically, the authors are encouraging further studies with relatively larger sample size.

## Conclusion

Endoscopic classic and reversal stapedotomies are comparable to each other as regards hearing outcome and surgical complications. The authors recommend further studies with relatively larger sample size.

## Data Availability

The datasets generated during and/or analysed during the current study are available from the corresponding author on reasonable request.
